# Molecular Characterization of *Leishmania RNA virus 2* in *Leishmania* *major* from Uzbekistan

**DOI:** 10.3390/genes10100830

**Published:** 2019-10-21

**Authors:** Yuliya Kleschenko, Danyil Grybchuk, Nadezhda S. Matveeva, Diego H. Macedo, Evgeny N. Ponirovsky, Alexander N. Lukashev, Vyacheslav Yurchenko

**Affiliations:** 1 Martsinovsky Institute of Medical Parasitology, Tropical and Vector Borne Diseases, Sechenov University, 119435 Moscow, Russia; ykleschenko@gmail.com (Y.K.); ucheb.mn@gmail.com (N.S.M.); eponirovsky@mail.ru (E.N.P.); alexander_lukashev@hotmail.com (A.N.L.); 2 Life Sciences Research Centre, Faculty of Science, University of Ostrava, 71000 Ostrava, Czech Republic; danilaman@gmail.com (D.G.); diegohqm@gmail.com (D.H.M.); 3 CEITEC—Central European Institute of Technology, Masaryk University, 62500 Brno, Czech Republic; 4 Department of Molecular Biology, Faculty of Biology, Moscow State University, 119991 Moscow, Russia

**Keywords:** *Leishmania RNA virus*, next-generation sequencing, LRV2

## Abstract

Here we report sequence and phylogenetic analysis of two new isolates of *Leishmania RNA virus 2* (LRV2) found in *Leishmania major* isolated from human patients with cutaneous leishmaniasis in south Uzbekistan. These new virus-infected flagellates were isolated in the same region of Uzbekistan and the viral sequences differed by only nineteen SNPs, all except one being silent mutations. Therefore, we concluded that they belong to a single LRV2 species. New viruses are closely related to the LRV2-Lmj-ASKH documented in Turkmenistan in 1995, which is congruent with their shared host (*L*. *major*) and common geographical origin.

## 1. Introduction

*Leishmaniavirus* is a genus of the family *Totiviridae*. In addition to this, the family includes similar viruses found in other protists (*Giardia*, *Trichomonas*, *Eimeria*) and various fungi [1]. Their virions are icosahedral (T = pseudo 2), non-enveloped, and approximately 40 nm in diameter [2,3,4]. The double-stranded RNA (dsRNA) genome of *Leishmaniavirus* is not segmented, is approximately 5.2 kb in length, and contains two open reading frames (ORFs) for the capsid protein and the RNA-dependent RNA polymerase (RDRP) [5,6]. The capsid ORF has a dedicated start-codon, whereas RDRP is translated as a C-terminal extension of the capsid. In different leishmania viruses, the mechanism of fusion Gag-Pol protein production varies, with RDRP located in +1 or -1 frameshift relative to or in-frame with the capsid [7,8].

Based on their phylogeny, leishmania viruses were divided into two major groups: *Leishmania RNA virus 1* (LRV1), infecting New World *Leishmania* (representatives of the subgenus *Viannia*: *L*. *guyanensis*, *L*. *braziliensis*) and LRV2, documented in the Old World leishmanias (representatives of the subgenus *Leishmania*: *L*. *major*, *L*. *aethiopica*, *L*. *infantum*). The first LRV2 was isolated from *L*. *major* in Turkmenistan [9]. The phylogenies of LRV1/2 and their respective hosts are congruent, suggesting the coevolution between the virus and its *Leishmania* host [8,10]. It was assumed that the entire lifecycle of totiviruses takes place in the cell cytoplasm and they never produce extracellular virions [1,11]. This view was supported by the coevolution pattern observed in LRVs. However, our recent study provided phylogenetic evidence for the LRV transfer between *Leishmania* and *Blechomonas*, two separate genera of the family Trypanosomatidae [12]. In addition, it has been recently demonstrated that LRV1 can exit the cell via extracellular vesicles, explaining the assumed cell-to-cell virus transfer [13].

LRV1 contributes to the pathogenicity of the New World *L. guyanensis* by interfering with the immune response of a vertebrate host [14]. Viral dsRNA interacts with the Toll-like receptor 3 (TLR3) inside parasitophorous vacuole, leading to the overexpression of pro-inflammatory cytokines (TNF-α, IL-6, INF-γ) and, subsequently, chronic inflammation in the primary lesion. This, in turn, facilitates the parasites’ dissemination to the secondary site (nasal mucosa), manifesting itself as a muco-cutaneous leishmaniasis (MCL) [15,16]. These crucial findings spurred the research on prevalence and diversity of *Leishmania* viruses [17], as well as viruses infecting other trypanosomatids [18,19]. The primary focus of this research was LRV1 in *Leishmania* (*Viannia*) spp. [20,21]. It was reported that LRV1 is more common in *Leishmania* isolates originating from Brazilian Amazonia, whereas isolates from the southern states of Brazil are mostly virus-free [21]. This implies that other factors, such as co-infection with other RNA viruses [22] or glycoconjugates [23], may also contribute to the *Viannia* pathogenicity.

The vast majority of the Old World leishmanias causes localized, slowly healing sores (cutaneous leishmaniasis (CL)). The notable exception is *Leishmania donovani*, which causes disseminated visceral leishmaniasis affecting many internal organs, such as the spleen, liver, and bone marrow [24]. Despite high pathogenicity, no LRVs were detected in 22 clinical isolates of *L*. *donovani*. However, 15 of these isolates contained another virus-infected trypanosomatid, *Leptomonas seymouri*, alongside *L*. *donovani* [25]. *Leptomonas seymouri* bears a capsid-less Narna-like virus and lacks RNA interference pathways, leading to accumulation of high amounts of dsRNA in the cytoplasm [26,27]. Thus, the influence of viral RNA on the development of symptoms, caused by *L*. *donovani*, is plausible. In *Leishmania aethiopica*, LRV2 was associated with elevated levels of TNF-α and IL-6 in vitro, suggesting that LRV1 and 2 may have a similar impact on the vertebrate immune system [8]. Besides originally described LRV2 from *L*. *major* in Turkmenistan and *L*. *aethiopica* in Ethiopia, a handful of virus positive isolates have been recently documented in Iran (*L*. *infantum* and *L*. *major*) [28] and Turkey (*L*. *major*) [29]. For these samples, only partial sequences were reported, precluding their robust phylogenetic analysis. In general, LRV2s appear to be less abundant compared to LRV1s.

In Uzbekistan, over 6000 cases of CL, caused by *Leishmania major*, were reported throughout the mid-20th century. Some of these cases were associated with a highly aggressive strain of *L*. *major* [30]. In the years following the collapse of the Soviet Union, the absence of appropriate preventive and monitoring activities led to the reemergence of leishmaniasis in Uzbekistan and other countries in Central Asia, making it a major public health problem in this region [31,32]. CL in Central Asia is mostly zoonotic, with gerbils serving as a natural reservoir of parasites [33,34]. In this study, we conducted a survey and sequence analysis of LRVs found in different *Leishmania* spp. isolated from both wild rodents and human patients.

## 2. Materials and Methods

### 2.1. Parasite Culture and RNA Isolation

Cryopreserved stocks of 10 *Leishmania* spp. isolates (3 *L. major*, 4 *L. turanica*, 2 *L. gerbilli*, and 1 *L. infantum*), collected from wild gerbils and lesions of infected patients in Central Asia, were initially grown on a biphasic blood agar overlaid with the M199 medium (Sigma–Aldrich, St. Louis, MO, USA) for 1 week. *Leishmania* promastigotes were then transferred to the M199 medium, supplemented with 10 mg/mL of hemin (Jena Bioschience Gmbh, Jena, Germany), 10% fetal bovine serum, 500 units/mL of penicillin, and 50 µg/mL of streptomycin (all from Thermo Fisher Scientific, Waltham, MA, USA) at 25 °C.

### 2.2. dsRNA Isolation and Next-Generation Sequencing

Total RNA extraction from 10^8^ promastigotes was performed using TRIzol isolation reagent (Thermo Fisher Scientific) as described previously [19]. Fifty mg of total RNA from each sample were treated with RNase-free DNase I (New England Biolabs, Ipswich, USA) and S1 nuclease from *Aspergillus oryzae* (Sigma–Aldrich) according to the manufacturer’s instructions. Viral dsRNA bands were visualized on 0.8% agarose gel and stained with ethidium bromide. Individual dsRNA bands were gel purified using a Zymoclean Gel RNA Recovery Kit (Zymo Research, Irvine, CA, USA). RiboMinus libraries were generated and sequenced using the Illumina NovaSeq platform (Illumina, San Diego, CA, USA) at Macrogen Inc. (Seoul, South Korea).

### 2.3. Bioinformatics Analysis

The raw sequence reads were trimmed with Trimmomatic v. 0.36 [35]. Read mapping was done in Bowtie2 v. 2.3.4.1 [36] and SAMtools v. 1.8 [37], and assembled de novo with Trinity [38]. Coverage was calculated using BEDTools v. 2.25 software [39]. ORFs of the assembled viral contigs were analyzed with NCBI ORFfinder [40]. The mutational analysis was accomplished in the HIVE-hexagon Population Analysis Tool program [41]. For the phylogenetic analyses, amino acid sequences of the capsid and RDRP were separately aligned by MAFFT v. 7.313 E-INS-i algorithm [42], trimmed with TrimAl v. 1.7 using “automated1” settings [43] and concatenated in FaBox v. 1.5 [44]. For both partitions, LG + F + I + G4 was selected as a best fit model by ModelFinder [45]. Maximum likelihood analysis with standard bootstrap method (1000 replicas) was performed in IQ-TREE v. 1.6.1 [46].

## 3. Results

**Figure 1:**
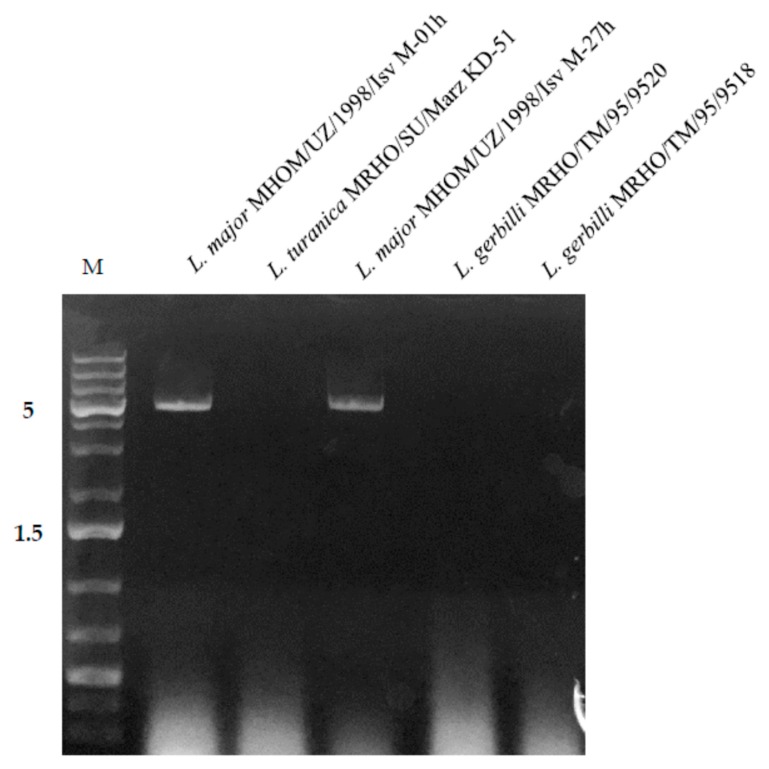
Detection of viral double-stranded RNA (dsRNA) on agarose gel. M; Gene Ruler 1 kb Plus ladder. Sizes on the left are in kb.

Ten *Leishmania* strains (*L*. *major*, *L*. *turanica*, *L*. *gerbilli*) isolated mostly from the CL-infected gerbils and human patients in different endemic areas of Turkmenistan, Kazakhstan, and Uzbekistan (Table 1) were screened for the presence dsRNA (indicating viral infection [19]). Two *L*. *major* isolates, both isolated form the soars of humans in Muborak, Qashqadaryo region in southern Uzbekistan, were found to be positive. Other analyzed samples from other districts of Uzbekistan (Karakalpak, Jambyl region, and Termez, Surxondaryo region), Turkmenistan (Serdar, Balkan region, and Tejen, Ahal region,) and Kazakhstan (Embi, Aktobe region) appeared negative on dsRNA gel electrophoresis. Both positive samples were characterized by a distinct 5.2 kbp band, which corresponded in size to the genomic dsRNA of the *Leishmania RNA virus* (Figure 1).

**Figure 2:**
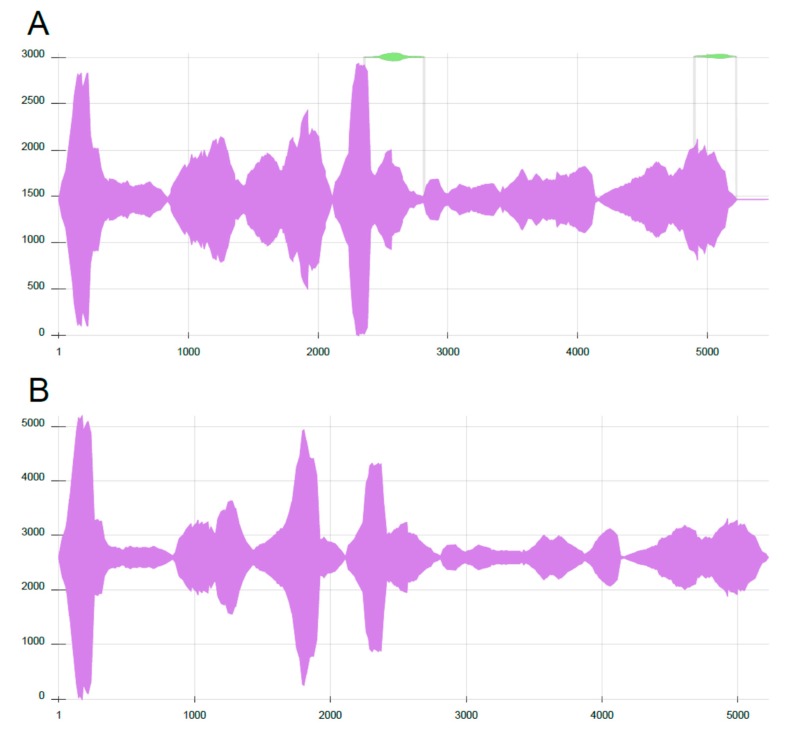
Clonal analysis revealed heterogeneous viral populations in LRV2-Lmj-Uzb1. Nucleotide positions are on the X axis, the Y axis serves as a ruler in the Sankey graph and represents depth of coverage. (**A**). LRV2-Lmj-Uzb1 has additionally two minor clones (green colour inserts). (**B**). LRV2-Lmj-Uzb2 is homogeneous.

RNA-sequencing was performed on positive samples and sequences were deposited to GenBank as LRV2-Lmj-Uzb1 and 2 (GenBank Acc. No. MN418974 and MN418975, respectively). Both sequences were 97% similar to the previously described LRV2-Lmj-ASKH isolated from *L*. *major* in Turkmenistan and had the same in-frame arrangement of capsid and RDRP ORFs with a single stop codon between the frames. Nucleotide sequences around the stop codon, where RNA pseudoknot and ribosome shunting sites were previously predicted [9], were invariably conserved in all three viral isolates. In-depth mutational analysis showed 170 nt/40 aa and 174 nt/41 aa differences between Uzb1/ASKH and Uzb2/ASKH sequences, respectively. In turn, Uzb1 and Uzb2 isolates varied by 19 SNPs, with only one resulting in amino acid substitution (T197I) in the RDRP (Table 2). Out of 19, there were three mutations with less than 100% frequencies in RNA-seq data, indicating that several viral subpopulations may be present in one isolate. Clonal analysis revealed heterogeneity in the Uzb1 isolate (Figure 2A), whereas the Uzb2 isolate was uniform (Figure 2B). The Uzb1 isolate showed two minor alternatively assembled regions, around 450 and 320 bp long (Figure 2A), which differed by 2 and 1 SNPs, respectively. Quantitatively, these alternative sequences accounted for 4.3% and 2.7% of the main contig abundance, respectively.

**Figure 3:**
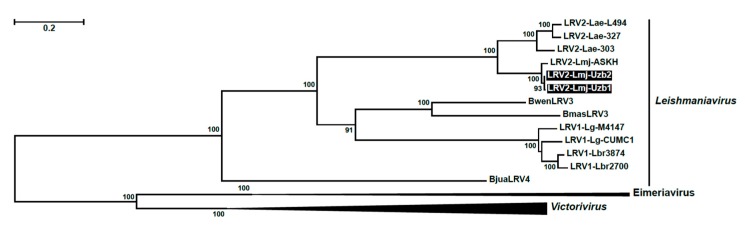
Maximum likelihood tree of LRVs built on amino acid sequence alignment using LG + F + I + G4 model. Newly identified isolates are highlighted in black. Standard bootstrap support (1000 replicas) values above 85 are shown. *Victorivirus* and *Eimeriavirus* (*Totiviridae*) were used as an outgroup. Scale bar represents the number of substitutions per site.

Maximum likelihood phylogenetic analysis positioned newly identified isolates sister to the LRV2-Lmj-ASKH with maximal statistical support, which correlates with their common geographical origin in Central Asia (Figure 3). Currently, three host-specific LRV lineages can be defined in *Leishmania*: LRV1 (New World clade), infecting *L*. *gyanensis* and *L*. *braziliensis*, LRV2 (Ethiopian clade) found in *L*. *aethiopica*, and LRV2 (Central Asian clade) from *L*. *major*. When comparing nucleotide sequences of Uzb1 and Uzb2 to the reference strain LRV2-Lmj-ASKH, we noticed that both isolates had atypical frame-shift regions in the capsid and RDRP ORFs. There were four such regions with the length of 51 bp (capsid), 23 bp, 28 bp, and 21 bp (RDRP) long (Figure S1). Importantly, closely related LRV sequences, belonging to the same clade, lack indels and differ only by SNPs. Large indels are starting to be apparent only when LRVs from different clades are compared, at which point sequence similarity drops below 68%. Visual examination of the frame-shift regions revealed the presence of the singleton indels in the LRV2-Lmj-ASKH sequence, which can be explained by the sequencing errors (it was analyzed in 1995 [9]).

## 4. Discussion

In this paper, we present sequence and phylogenetic analysis of two newly identified leishmania viruses (LRV2) of *Leishmania major* isolated from human CL patients in southern Uzbekistan. This is the second report of LRV2 in *L*. *major* in Central Asia, following the original description of LRV2-Lmj-ASKH isolate from Turkmenistan in 1995 [9]. Two viruses were documented in the same host species (*L. major*) in the same geographical region, arguing that they may represent just one viral species.

Phylogenetic and mutational analyses suggest that LRV2-Lmj-Uzb1 and LRV2-Lmj-Uzb2 were most closely related to each other and the previously described LRV2-Lmj-ASKH. The phylogenetic position of these viruses correlates with their common geographical origin in Central Asia. Also of note, the leishmanian LRV1 and LRV2, infecting the same or closely related species of trypanosomatids, are monophyletic. Together, these observations support the currently prevailing view of predominantly vertical transmission and tight coevolution of the virus with its *Leishmania* spp. host [8,10]. Assembly of the RNA-seq data of the Uzb1 isolate yielded alternative contigs with the abundance 20–30 times lower than that of the major contig. This indicates the presence of the viral subpopulation in the Uzb1 sample. Given the low proportion and small number of mutations, it is parsimonious to suggest that the observed minor sequences are the result of clonal evolution of the virus. To date, it appears that the “tight coevolution” rule is applicable only to LRVs infecting *Leishmania*. The recent finding of LRV3 and 4 in *Blechomonas* spp. [12] suggests that there were at least two events of horizontal viral transfers between *Blechomonas* and *Leishmania* spp. In general, horizontal viral transfers are quite common within monoxenous trypanosomatids, with arthropods serving as mediators of viral exchange [19]. It is possible that the transition of *Leishmania* to a dixenous lifecycle, which necessitated adaptation to a specific arthropod vector [47,48], isolated these flagellates from their monoxenous kin. This, in turn, has cut down the viral flow between *Leishmania* and other trypanosomatids, resulting in the strict coevolutionary patterns observed nowadays.

Our analysis revealed only two positive isolates out of 10 samples analyzed, which is consistent with the overall low prevalence of *Leishmaniavirus* in the Old World leishmanias [16]. In other studies, only 2 LRV-positive isolates (one *L*. *infantum* and one *L*. *major*) were documented out of 50 tested in Iran [28] and no LRV-positive *L*. *donovani* isolates were found among 22 tested in India [25]. Old World *Leishmania* spp. do not cause MCL, in which LRV1 was shown to be involved [14,16]. On the other hand, LRV2 from *L*. *aethiopica* was found to display similar immunological effects as LRV1 in vitro [8]. Thus, LRV2 might influence development of the visceral disease, which is a predominant form of disseminated leishmaniasis in the Old World. Currently, there is only one report of LRV2 in *L*. *infantum* isolated from a visceral leishmaniasis patient, but the potential role of this virus in etiology and progression of the disease was not investigated [28]. Studies of this kind are further complicated by the absence of isogenic virus-free isolates for Old World leishmanias [49]. Furthermore, it might be difficult to obtain such isolates as these parasites do not have a functional RNA-interference pathway [50,51] that was harnessed for curing LRV1 in *L*. *guyaynensis* [52]. Consequently, broader sampling and more in-depth molecular studies are needed to elucidate the diversity of LRV viruses, as well as their interaction with *Leishmania* and vertebrate hosts.

## Figures and Tables

**Table 1:** *Leishmania* spp. analyzed in this work.

*Leishmania* spp.	WHO Code	LRV2	Source ^1^	Origin
*L. turanica*	MRHO/KZ/87/MARZBK7	-	*R. opimus*	Embi, Aktobe region, Kazakhstan
*L. major*	MHOM/UZ/1998/Isv M-01h	+	*H. sapiens*	Muborak, Qashqadaryo region, Uzbekistan
*L. turanica*	MRHO/UZ/87MarzKK-52R	-	*R. opimus*	Karakalpak, Jambyl region, Uzbekistan
*L. turanica*	MRHO/SU/Marz KD-51	-	*R. opimus*	Karakalpak, Jambyl region, Uzbekistan
*L. gerbilli*	MRHO/TM/95/9520	-	*R. opimus*	Serdar, Balkan region, Turkmenistan
*L. gerbilli*	MRHO/TM/95/9518	-	*R. opimus*	Serdar, Balkan region, Turkmenistan
*L. major*	MHOM/UZ/1998/Isv M-27h	+	*H. sapiens*	Muborak, Qashqadaryo region, Uzbekistan
*L. infantum*	MHOM/KZ/75/MarzDzha	-	*H. sapiens*	Karakalpak, Jambyl region, Uzbekistan
*L. turanica*	I/TM/95/Ph-82	-	*P. papatasi*	Tejen, Ahal region, Turkmenistan
*L. major*	MRHO/UZ/2003/Isv T-38g	-	*R. opimus*	Termez, Surxondaryo region, Uzbekistan

^1^ Species are abbreviated as follows: *R. opimus* is *Rhombomys opimus* (great gerbil), H. *sapiens* is *Homo sapiens*, *P. papatasi* is *Phlebotomus papatasi* (sandfly), LRV2 is *Leishmania RNA virus 2*. “+” and “-” denote presence and absence, respectively.

**Table 2:** SNP comparison between LRV2-Lmj-Uzb1 and LRV2-Lmj-Uzb2. Coverage indicates a total number of sequence reads covering each position on the genome.

Position, nt	LRV2-Lmj-Uzb1	LRV2-Lmj-Uzb1	Frequency, %	Coverage
389	C	T	100	384
539	T	A	40	372
1181	C	A	100	1525
1415	T	C	52	430
1517	C	T	100	732
1772	A	G	100	3616
1835	A	G	100	4188
1892	G	A	100	3890
1922	T	G	100	1434
2603	T	A	100	836
2648	G	A	100	593
3064	T	C	100	345
3125	G	A	100	493
3434	A	G	31	224
3464	A	G	100	289
3806	A	T	100	698
3809	G	A	100	686
3881	G	A	100	704
4892	C	T	100	1154

## Supplementary Materials

The following are available online at https://www.mdpi.com/2073-4425/10/10/830/s1, Figure S1: Singleton indels in LRV2-Lmj-ASKH (U32108.1) resulting in frame shifts. A. Nucleotide sequences of three clades of *Leishmania* LRVs (LRV1, LRV2-Lae, and LRV-Lmj) were aligned using MAFFT Eins-i and visually inspected in BioEdit. Alignment columns with ≥50% similarity in analyzed species are colored. Nucleotide positions, which correspond to insertions in LRV2-Lmj-ASKH sequence, are numbered. In cases of deletion, two consecutive nucleotide positions are numbered B. Amino acid sequence alignment of LRV2-Lmj-ASKH and LRV2-Lm-Uzb1 showing the frame-shifted regions corresponding to nucleotide sequences shown in panel A.

## References

[1] Ghabrial S.A., Castón J.R., Jiang D., Nibert M.L., Suzuki N. (2015). 50-plus years of fungal viruses. Virology.

[2] Janssen M.E., Takagi Y., Parent K.N., Cardone G., Nibert M.L., Baker T.S. (2015). Three-dimensional structure of a protozoal double-stranded RNA virus that infects the enteric pathogen *Giardia lamblia*. J. Virol..

[3] Parent K.N., Takagi Y., Cardone G., Olson N.H., Ericsson M., Yang M., Lee Y., Asara J.M., Fichorova R.N., Baker T.S. (2013). Structure of a protozoan virus from the human genitourinary parasite Trichomonas vaginalis. MBio.

[4] Dunn S.E., Li H., Cardone G., Nibert M.L., Ghabrial S.A., Baker T.S. (2013). Three-dimensional structure of victorivirus HvV190S suggests coat proteins in most totiviruses share a conserved core. PLoS Pathog..

[5] Stuart K.D., Weeks R.L., Guilbride P.J. (1992). Myler molecular organization of *Leishmania RNA virus 1*. Proc. Natl. Acad. Sci. USA.

[6] Scheffter S., Widmer G., Patterson J.L. (1994). Complete sequence of *Leishmania RNA virus 1*–*4* and identification of conserved sequences. Virology.

[7] Lee S.E., Suh J.M., Scheffter S., Patterson J.L., Chung I.K. (1996). Identification of a ribosomal frameshift in *Leishmania RNA virus* 1–4. J. Biochem..

[8] Zangger H., Hailu A., Desponds C., Lye L.F., Akopyants N.S., Dobson D.E., Ronet C., Ghalib H., Beverley S.M., Fasel N. (2014). *Leishmania aethiopica* field isolates bearing an endosymbiontic dsRNA virus induce pro-inflammatory cytokine response. PLoS Negl. Trop. Dis..

[9] Scheffter S.M., Ro Y.T., Chung I.K., Patterson J.L. (1995). The complete sequence of *Leishmania RNA virus* LRV2-1, a virus of an Old World parasite strain. Virology.

[10] Widmer G., Dooley S. (1995). Phylogenetic analysis of *Leishmania RNA virus* and *Leishmania* suggests ancient virus-parasite association. Nucleic Acids Res..

[11] Okamoto K., Miyazaki N., Larsson D.S., Kobayashi D., Svenda M., Muhlig K., Maia F.R., Gunn L.H., Isawa H., Kobayashi M. (2016). The infectious particle of insect-borne totivirus-like *Omono River virus* has raised ridges and lacks fibre complexes. Sci. Rep..

[12] Grybchuk D., Kostygov A.Y., Macedo D.H., Votypka J., Lukes J., Yurchenko V. (2018). RNA viruses in Blechomonas (Trypanosomatidae) and evolution of Leishmaniavirus. MBio.

[13] Atayde V.D., da Silva A., Filho L., Chaparro V., Zimmermann A., Martel C., Jaramillo M., Olivier M. (2019). Exploitation of the *Leishmania* exosomal pathway by *Leishmania RNA virus 1*. Nat. Microbiol..

[14] Ives A., Ronet C., Prevel F., Ruzzante G., Fuertes-Marraco S., Schutz F., Zangger H., Revaz-Breton M., Lye L.F., Hickerson S.M. (2011). *Leishmania* RNA virus controls the severity of mucocutaneous leishmaniasis. Science.

[15] Hartley M.A., Drexler S., Ronet C., Beverley S.M., Fasel N. (2014). The immunological, environmental, and phylogenetic perpetrators of metastatic leishmaniasis. Trends Parasitol..

[16] Hartley M.A., Ronet C., Zangger H., Beverley S.M., Fasel N. (2012). Leishmania RNA virus: When the host pays the toll. Front. Cell Infect Microbiol..

[17] Tirera S., Ginouves M., Donato D., Caballero I.S., Bouchier C., Lavergne A., Bourreau E., Mosnier E., Vantilcke V., Couppie P. (2017). Unraveling the genetic diversity and phylogeny of *Leishmania RNA virus 1* strains of infected *Leishmania* isolates circulating in French Guiana. PLoS Negl. Trop. Dis..

[18] Grybchuk D., Kostygov A.Y., Macedo D.H., d’Avila-Levy C.M., Yurchenko V. (2018). RNA viruses in trypanosomatid parasites: A historical overview. Mem. Inst. Oswaldo Cruz.

[19] Grybchuk D., Akopyants N.S., Kostygov A.Y., Konovalovas A., Lye L.F., Dobson D.E., Zangger H., Fasel N., Butenko A., Frolov A.O. (2018). Viral discovery and diversity in trypanosomatid protozoa with a focus on relatives of the human parasite Leishmania. Proc. Natl. Acad. Sci. USA..

[20] Adaui V., Lye L.F., Akopyants N.S., Zimic M., Llanos-Cuentas A., Garcia L., Maes I., De Doncker S., Dobson D.E., Arevalo J. (2016). Association of the endobiont double-stranded RNA virus LRV1 with treatment failure for human leishmaniasis caused by *Leishmania braziliensis* in Peru and Bolivia. J. Infect. Dis..

[21] Ginouvès M., Simon S., Bourreau E., Lacoste V., Ronet C., Couppie P., Nacher M., Demar M., Prevot G. (2016). Prevalence and distribution of *Leishmania RNA Virus 1* in *Leishmania* parasites from French Guiana. Am. J. Trop. Med. Hyg..

[22] Rossi M., Castiglioni P., Hartley M.A., Eren R.O., Prevel F., Desponds C., Utzschneider D.T., Zehn D., Cusi M.G., Kuhlmann F.M. (2017). Type I interferons induced by endogenous or exogenous viral infections promote metastasis and relapse of leishmaniasis. Proc. Natl. Acad. Sci. USA..

[23] Paranaiba L.F., Pinheiro L.J., Macedo D.H., Menezes-Neto A., Torrecilhas A.C., Tafuri W.L., Soares R.P. (2018). An overview on *Leishmania* (*Mundinia*) *enriettii*: Biology, immunopathology, LRV and extracellular vesicles during the host-parasite interaction. Parasitology.

[24] Bruschi F., Gradoni L. (2018). The Leishmaniases: Old Neglected Tropical Diseases.

[25] Sukla S., Roy S., Sundar S., Biswas S. (2017). *Leptomonas seymouri narna-like virus 1* and not leishmaniaviruses detected in kala-azar samples from India. Arch. Virol..

[26] Kraeva N., Butenko A., Hlaváčová J., Kostygov A., Myškova J., Grybchuk D., Leštinová T., Votýpka J., Volf P., Opperdoes F. (2015). *Leptomonas seymouri*: Adaptations to the dixenous life cycle analyzed by genome sequencing, transcriptome profiling and co-infection with *Leishmania donovani*. PLoS Pathog..

[27] Lye L.F., Akopyants N.S., Dobson D.E., Beverley S.M. (2016). A *Narnavirus*-like element from the trypanosomatid protozoan parasite *Leptomonas seymouri*. Genome Announc..

[28] Hajjaran H., Mahdi M., Mohebali M., Samimi-Rad K., Ataei-Pirkooh A., Kazemi-Rad E., Naddaf S.R., Raoofian R. (2016). Detection and molecular identification of *Leishmania RNA virus* (LRV) in Iranian *Leishmania* species. Arch. Virol..

[29] Kurt O., Mansur N., Cavus I., Ozcan O., Batir M.B., Gunduz C., Sezerman O.U., Ozbilgin A. (2019). First report and in silico analysis of *Leishmania virus* (LRV2) identified in an autochthonous *Leishmania major* isolate in Turkey. New Microbiol..

[30] Faulde M.K., Werner A., Heyl G. (2007). Untreated zoonotic cutaneous leishmaniasis characterizing a highly aggressive strain type of *Leishmania major* in Uzbekistan. J. Eur. Acad. Dermatol. Venereol..

[31] Zhirenkina E.N., Ponirovskii E.N., Strelkova M.V., Morozov E.N., Flegontov P.N., Kolesnikov A.A., Ponomareva V.I., Nasyrova R.M., Kovalenko D.A., Fatullaeva A.A. (2011). The epidemiological features of visceral leishmaniasis, revealed on examination of children by Polymerase Chain Reaction, in the Papsky District, Namangan Region, Uzbekistan. Med. Parazitol. Parazit. Bolezn..

[32] Strelkova M.V., Ponirovsky E.N., Morozov E.N., Zhirenkina E.N., Razakov S.A., Kovalenko D.A., Schnur L.F., Schonian G. (2015). A narrative review of visceral leishmaniasis in Armenia, Azerbaijan, Georgia, Kazakhstan, Kyrgyzstan, Tajikistan, Turkmenistan, Uzbekistan, the Crimean Peninsula and Southern Russia. Parasit. Vectors.

[33] Chajbullinova A., Votýpka J., Sádlová J., Kvapilová K., Seblová V., Kreisinger J., Jirků M., Sanjoba C., Gantuya S., Matsumoto Y. (2012). The development of *Leishmania turanica* in sand flies and competition with *L. major*. Parasit. Vectors.

[34] Akhoundi M., Kuhls K., Cannet A., Votýpka J., Marty P., Delaunay P., Sereno D. (2016). A historical overview of the classification, evolution, and dispersion of *Leishmania* parasites and sandflies. PLoS Negl. Trop. Dis..

[35] Bolger A.M., Lohse M., Usadel B. (2014). Trimmomatic: A flexible trimmer for Illumina sequence data. Bioinformatics.

[36] Langmead B., Salzberg S.L. (2012). Fast gapped-read alignment with Bowtie 2. Nat. Methods.

[37] Li H., Handsaker B., Wysoker A., Fennell T., Ruan J., Homer N., Marth G., Abecasis G., Durbin R.S. (2009). Genome Project Data Processing The Sequence Alignment/Map format and SAMtools. Bioinformatics.

[38] Grabherr M.G., Haas B.J., Yassour M., Levin J.Z., Thompson D.A., Amit I., Adiconis X., Fan L., Raychowdhury R., Zeng Q. (2011). Full-length transcriptome assembly from RNA-Seq data without a reference genome. Nat. Biotechnol..

[39] Quinlan A.R. (2014). BEDTools: The swiss-army tool for genome feature analysis. Curr. Protoc. Bioinform..

[40] Wheeler D.L., Barrett T., Benson D.A., Bryant S.H., Canese K., Chetvernin V., Church D.M., Dicuccio M., Edgar R., Federhen S. (2008). Database resources of the National Center for Biotechnology Information. Nucleic Acids Res..

[41] Karagiannis K., Simonyan V., Chumakov K., Mazumder R. (2017). Separation and assembly of deep sequencing data into discrete sub-population genomes. Nucleic Acids Res..

[42] Katoh K., Standley D.M. (2013). MAFFT multiple sequence alignment software version 7: Improvements in performance and usability. Mol. Biol. Evol..

[43] Capella-Gutiérrez S., Silla-Martinez J.M., Gabaldon T. (2009). trimAl: A tool for automated alignment trimming in large-scale phylogenetic analyses. Bioinformatics.

[44] Villesen P. (2007). FaBox: An online toolbox for fasta sequences. Mol. Ecol. Notes.

[45] Kalyaanamoorthy S., Minh B.Q., Wong T.K.F., von Haeseler A., Jermiin L.S. (2017). ModelFinder: Fast model selection for accurate phylogenetic estimates. Nat. Methods.

[46] Nguyen L.T., Schmidt H.A., von Haeseler A., Minh B.Q. (2015). IQ-TREE: A fast and effective stochastic algorithm for estimating maximum-likelihood phylogenies. Mol. Biol. Evol..

[47] Lukeš J., Butenko A., Hashimi H., Maslov D.A., Votýpka J., Yurchenko V. (2018). Trypanosomatids are much more than just trypanosomes: Clues from the expanded family tree. Trends Parasitol..

[48] Lukeš J., Skalický T., Týč J., Votýpka J., Yurchenko V. (2014). Evolution of parasitism in kinetoplastid flagellates. Mol. Biochem. Parasitol..

[49] Zangger H., Ronet C., Desponds C., Kuhlmann F.M., Robinson J., Hartley M.A., Prevel F., Castiglioni P., Pratlong F., Bastien P. (2013). Detection of Leishmania RNA virus in *Leishmania* parasites. PLoS Negl. Trop. Dis..

[50] Lye L.F., Owens K., Shi H., Murta S.M., Vieira A.C., Turco S.J., Tschudi C., Ullu E., Beverley S.M. (2010). Retention and loss of RNA interference pathways in trypanosomatid protozoans. PLoS Pathog..

[51] Matveyev A.V., Alves J.M., Serrano M.G., Lee V., Lara A.M., Barton W.A., Costa-Martins A.G., Beverley S.M., Camargo E.P., Teixeira M.M. (2017). The evolutionary loss of RNAi key determinants in kinetoplastids as a multiple sporadic phenomenon. J. Mol. Evol..

[52] Brettmann E.A., Shaik J.S., Zangger H., Lye L.F., Kuhlmann F.M., Akopyants N.S., Oschwald D.M., Owens K.L., Hickerson S.M., Ronet C. (2016). Tilting the balance between RNA interference and replication eradicates Leishmania RNA virus 1 and mitigates the inflammatory response. Proc. Natl. Acad. Sci. USA.

