# Supplementary material for: Molecular Characterization of Leishmania RNA virus 2 in Leishmania major from Uzbekistan
Source: Genes (Basel). 2019 Oct 21;10(10):830. doi: 10.3390/genes10100830 (PMC6826456; doi:10.3390/genes10100830)
Supplement: Supplementary file 1 [file genes-10-00830-s001.pdf]

A

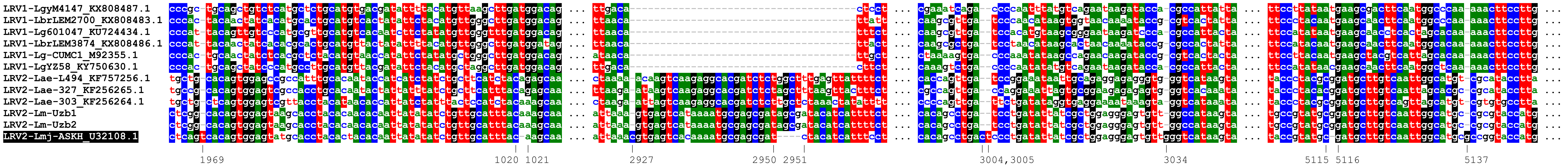

B

**LRV2-Lm-Uzb1**	capsid 205	PALNSA--QWSKHLHNTIIYLLHLQSNGDG	232	RDRP 143	NARIKVSHKMRAIAIHHFLE	162		RDRP 132	VVEGIDIPQPDPDIIA-GGSVGHKYK	188		RDRP 873	VLPYADACQLACRVPCNG	890	
		PALNS +++ LHN I + SNGDG			NARIK A +HHFLE				VVEGI IPQPD I++ G +GHKYK				VLPYA VPCNG		
**LRV2-Lmj-ASKH**	capsid 205	PALNSVTVEYAPTLHNYIS--VAFTSNGDG	232	RDRP 19	NARIK-RESQNASDLHHFLE	37		RDRP 39	VVEGIAIPQPDSLILSLEGVLGHKYK	64		RDRP 749	VLPYAMLVNWHAPVPCNG	766	
